# Effects of diabetes and hypertension on macrophage infiltration and matrix expansion in the rat kidney

**DOI:** 10.1186/1471-2369-6-6

**Published:** 2005-05-27

**Authors:** Andrea Hartner, Roland Veelken, Michael Wittmann, Nada Cordasic, Karl F Hilgers

**Affiliations:** 1 Children and Youth Hospital, University of Erlangen-Nuremberg, Loschgestrasse 15, D-91054 Erlangen, Germany; 2 Nephrology and Hypertension, University of Erlangen-Nuremberg, Loschgestrasse 8, D-91054 Erlangen, Germany; 3 Medicine II, Augsburg City Hospital, Stenglinstrasse 2, D-86156 Augsburg, Germany

## Abstract

**Background:**

In experimental models of diabetes mellitus, aggravation of renal injury by concomitant hypertension has been described. Inflammatory mechanisms contribute to renal damage in both diseases. We investigated whether hypertension and diabetes mellitus act synergistically to induce macrophage infiltration and matrix expansion in the kidney.

**Methods:**

Insulin-dependent diabetes mellitus was induced by streptozotocin injections to hypertensive mRen2-transgenic rats (TGR) and normotensive Sprague-Dawley control rats. Quantitative immunohistochemical examination of kidney tissue sections was used to measure macrophage infiltration and matrix expansion. The expression of MCP-1, Osteopontin, RANTES, ICAM-1 and VCAM-1 was evaluated by real-time RT-PCR. The localization of MCP-1 was studied by immunohistochemistry.

**Results:**

Macrophage infiltration was present in the kidney of normotensive diabetic rats. Hypertensive rats exhibited a more marked infiltration of macrophages, regardless of whether diabetes was present or not. Gene expression of ICAM-1, VCAM-1 and RANTES was unaltered whereas Osteopontin and MCP-1 were induced by hypertension. Immunoreactive MCP-1 was slightly increased in diabetic rat kidney podocytes, and more markedly increased in hypertensive animals. Glomerular matrix accumulation was induced by diabetes and hypertension to a similar degree, and was highest in hypertensive, diabetic animals.

**Conclusion:**

Diabetes mellitus caused a mild, and angiotensin-dependent hypertension a more marked infiltration of macrophages in the kidney. Combination of both diseases led to additive effects on matrix expansion but not on inflammation. Hypertension appears to be a much stronger stimulus for inflammation of the kidney than STZ diabetes, at least in mRen2-transgenic rats.

## Background

Diabetic nephropathy is the most common cause of end-stage renal failure in developed countries and its incidence continues to rise [1]. In most patients with diabetic nephropathy, hypertension is present and contributes significantly to the progression of renal failure in diabetes [1]. Studies in diabetic rats as well as in human volunteers with hyperglycemia indicated that activation of the intrarenal renin-angiotensin system (RAS) plays a key role in the development of the hemodynamic abnormalities in early diabetic nephropathy [2]. Angiotensin II-induced hypertension leads to macrophage infiltration in the kidney, and chemokines have been proposed as mediators of macrophage infiltration [3,4]. For example, the chemokine monocyte chemoattractant protein-1 (MCP-1) can be induced in vascular smooth muscle cells by angiotensin II [5]. The finding that MCP-1 is likewise induced in renal mesangial cells by high glucose concentrations [6] is in keeping with the hypothesis that chronic inflammatory mechanisms may also contribute to the pathogenesis of diabetic nephropathy [7]. Thus, induction of chemokines in the diabetic kidney seems to enhance macrophage infiltration [8-12].

Kelly et al. described that induction of osteopontin is related to macrophage infiltration in streptozotocin diabetic, mRen-2 transgenic hypertensive rats [13]. These authors had previously reported that this animal model resembles aspects of human diabetic nephropathy [14]. We used this animal model to examine the potential contribution of other chemokines (MCP-1, RANTES) and adhesion molecules to macrophage infiltration. Further, we sought to delineate the relative contribution of angiotensin-dependent hypertension from that of diabetes to inflammation by including normotensive diabetic rats of the same genetic background.

## Methods

### Rat models of hypertension and diabetes mellitus

Rats were housed in a room maintained at 22 ± 2°C, exposed to a 12 hour dark/light cycle. The animals were allowed unlimited access to chow (#1320, Altromin, Lage, Germany) and tap water. All procedures performed on animals were done in accordance with guidelines of the American Physiological Society and were approved by the local government authorities (Regierung von Mittelfranken, AZ # 621-2531.31-19/96). Eighteen male rats heterozygous for the mouse ren-2 transgene (TGR) with angiotensin II dependent hypertension [15] and 18 age-matched Sprague-Dawley-Hannover (SD) controls (Möllegaard, Eijby, Denmark) at an average body weight of 250 g were used for induction of diabetes by intraperitoneal injection of streptozotocin (STZ) (Sigma, Deisenhofen, Germany) (70 mg per kg of body weight) dissolved in 0.1 M sodium citrate buffer (pH 4.5). Two days later, blood was obtained from the tail vein and diabetes was confirmed by measurement of blood glucose using a reflectance meter (Glucometer Elite II, Bayer, Leverkusen, Germany). Only rats with a consistent blood glucose > 250 mg/dl were included (13 TGR and 12 SD). Diabetic and control rats were followed for 5 weeks. Blood glucose and systolic blood pressure (as measured by tail-cuff plethysmography under light ether anesthesia) were monitored weekly (at 8 a.m.). After five weeks, the rats were kept in metabolic cages for determination of urinary albumin excretion (enzyme immunoassay kit, CellTrend, Luckenwalde, Germany) for 24 hours. Subsequently, the rats were equipped with a femoral catheter and arterial blood pressure was measured via transducers (Grass Instruments, Quincy, USA) connected to a polygraph (Hellige, Freiburg, Germany) four hours after termination of anesthesia. Rats were sacrificed and kidneys were weighed and decapsulated. Half of each kidney was immediately snap-frozen in liquid nitrogen for later protein and RNA extraction. The other half was fixed in methyl-Carnoy solution (60% methanol, 30% chloroform and 10% glacial acetic acid) for histology and immunohistochemistry.

### Real-time RT-PCR detection of mRNA

Renal cortical tissue extraction and real-time RT-PCR were carried out as described [16]. Briefly, first-strand cDNA was synthesized with TaqMan reverse transcription reagents (Applied Biosystems, Darmstadt, Germany) using random hexamers as primers. Final RNA concentration in the reaction mixture was adjusted to 0,5 ng/μL. Reactions without Multiscribe reverse transcriptase were used as negative controls for genomic DNA contamination. PCR was performed with an ABI PRISM 7000 Sequence Detector System and TaqMan or SYBR Green Universal PCR Master Mix (Applied Biosystems) according to the manufacturers instructions. All samples were run in triplicates. The relative amount of the specific mRNA was normalized with respect to 18S rRNA. Primer design was accomplished with PrimerExpress software (Applied Biosystems). Primer sequences used are as follows. RANTES forward primer: GTCGTCTTTGTCACTCGAAGGA, RANTES reverse primer: GATGTATTCTTGAACCCACTTCTTCTC and RANTES probe: CCGCCAAGTGTGTG CCAACCC. ICAM-1 forward primer: GGGCCCCCTACCTTAGGAA, ICAM-1 reverse primer: GGGACAGTGTCCCAGCTTTC. VCAM-1 forward primer: TGTGGAAGTGT GCCCGAAAT, VCAM-1 reverse primer: TGCCTTGCGGATGGTGTAC. Primers and probes for 18S, MCP-1 and osteopontin were previously described [16-18].

### Western blot detection of MCP-1

Protein was extracted from kidneys of 5 rats of each group using Tri-reagent (MRC Inc.). Protein concentration was determined using a protein assay kit (Pierce, Rockford, IL, USA). Western blot analysis was performed as described before [3] with a polyclonal rabbit anti-rat MCP-1 antiserum which was kindly provided by Dr. T. Yoshimura, Frederick, MD and used at a dilution of 1:250.

### Immunohistochemistry

After overnight fixation in methyl-Carnoy solution, tissues were dehydrated by bathing in increasing concentrations of methanol, followed by 100% iso-propanol. After embedding in paraffin, 3 μm sections were cut with a Leitz SM 2000 R microtome (Leica Instruments, Nussloch, Germany). After deparaffinization, endogenous peroxidase activity was blocked with 3% H_2_O_2 _in methanol for 20 min at room temperature. Immunohistochemical detection of ED-1 was carried out as described [3]. The mouse monoclonal antibody against the macrophage marker ED-1 was purchased from Serotec (Biozol, Eching, Germany) and used at a dilution of 1:250. Renal cortical collagen I was detected by a rabbit polyclonal antibody (Biogenesis, Poole, England, UK) at a dilution of 1:1000. A goat polyclonal antibody to collagen IV (Southern Biotechnology Associates, Birmingham, AL, USA) was used at a dilution of 1:500. Each slide was counterstained with hematoxylin.

### Analysis of data

Intraglomerular ED-1 positive cells were counted in all glomeruli of a given kidney section (100–300 glomeruli, no selection) and expressed as cells per glomerular section. Interstitial ED-1 positive cells were counted in 30 medium-power (magnification 250 ×) cortical views per section and expressed as cells per square mm. Counting was begun in a random cortical field and in consecutive non-overlapping cortical fields to the right of the previous view without selection; if necessary, counting was continued at the opposite (left) edge of the section. MCP-1 staining was evaluated in >100 glomeruli and 20 cortical interstitial low-power (100 ×) fields by a blinded observer. Glomeruli were classified as showing no staining (score 0), staining of up to one third of the glomerular tuft area (score 1), staining affecting one to two thirds (score 2) or more than two thirds of the glomerular tuft (score 3). Interstitial fields were classified as showing no staining (score 0), one area of peritubular interstitial staining not spanning the circumference of a tubular cross-section (score 1); two such areas or one area of staining spanning the circumference of a tubular cross-section (score 2), peritubular staining involving less than 4 (score 3) or more than 4 (score 4) tubular cross-sections. Expansion of interstitial collagen I was measured by Metaview software (Visitron Systems, Puchheim, Germany) in 10 non-overlapping medium-power cortical views per section excluding glomeruli and was expressed as percent of stained area per cross section. Glomerular collagen IV staining was measured by Metaview in every third glomerulus per cross section, and the stained area was expressed as percentage of the total area of the glomerular tuft.

Two-way analysis of variance, followed by the post-hoc Bonferroni test with adjustment for multiple comparison, was used to compare groups. A p value < 0.05 was considered significant. The procedures were carried out using the SPSS version 11.5 software (SPSS Inc., Chicago, IL, USA). Values are displayed as means ± standard error of the mean.

## Results

**Figure 1:**
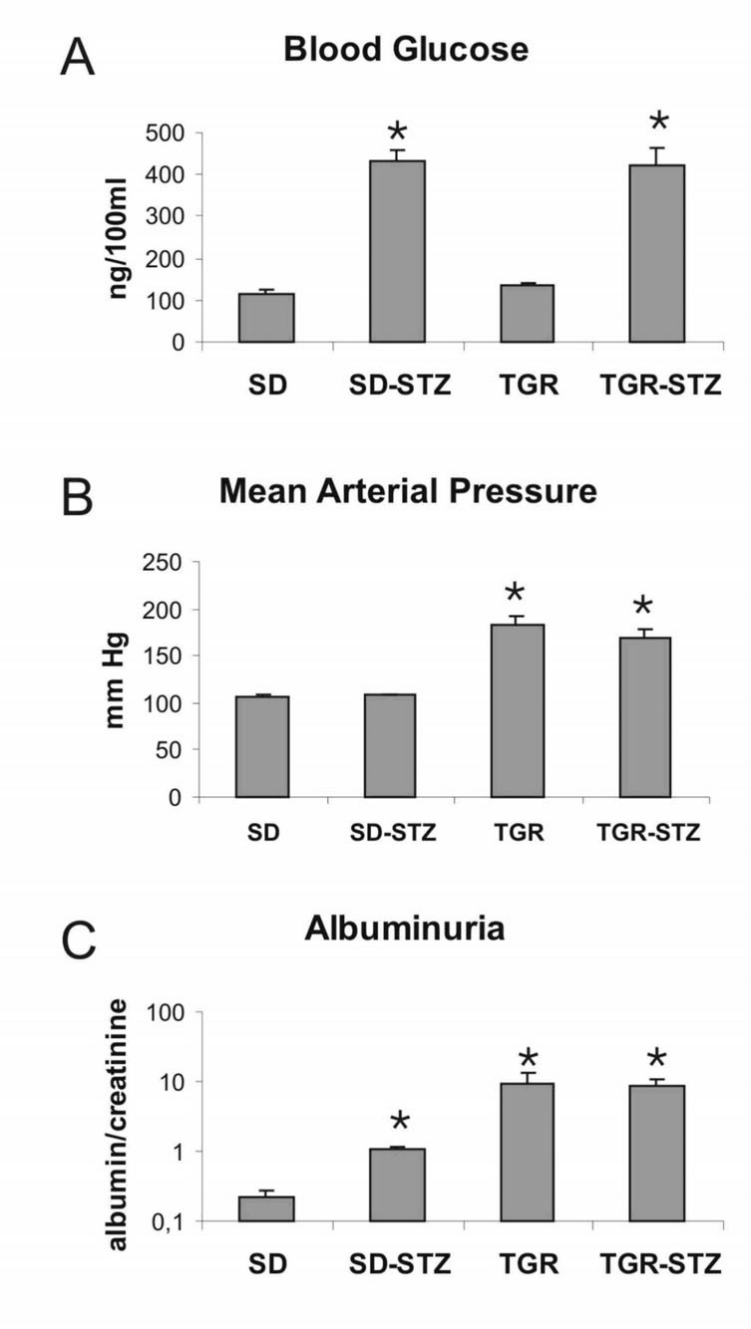
Blood glucose (panel A), arterial blood pressure (panel B) and albuminuria (panel C) of diabetic and hypertensive rats. Blood glucose was measured 24 hours before termination of the experiment. Mean arterial blood pressure was determined in awake rats via indwelling catheters inserted into the femoral artery. For determination of albuminuria, urine was collected 24 hours before sacrifice. SD, normotensive normoglycemic control rats; TGR, transgenic hypertensive rats; STZ, streptozotocin treatment. * indicates p < 0.05 versus normotensive normoglycemic SD control rats.

Injection of STZ induced diabetes mellitus equally well in SD and in TGR. In STZ-treated animals, blood glucose levels were not different between TGR and SD throughout the development of the disease (see figure 1A). In untreated TGR, blood glucose did not differ from untreated SD rats (figure 1A). Systolic and mean arterial blood pressure were significantly higher in TGR compared to SD (figure 1B). Development of hypertension was not affected by STZ treatment; there were no differences between STZ-treated and untreated control animals with regard to systolic blood pressure (data not shown) and mean intraarterial blood pressure measurements (figure 1B). Albumin excretion was increased in diabetic animals and even more in hypertensive rats; combination of both diseases did not further elevate albuminuria (figure 1C).

STZ diabetes led to reduced body weight gain and kidney hypertrophy. Kidney weight/body weight ratio was significantly increased in SD-STZ, TGR and TGR-STZ versus SD controls as well as in TGR-STZ versus TGR (table 1). TGR hypertensive animals had significantly higher heart weight/body weight ratios than normotensive SD rats, which was not affected by STZ diabetes (table 1). Diabetes led to polyuria (table 1), which was further increased in hypertensive rats. Urine production was slightly higher in TGR than in SD, but significantly elevated in the diabetic groups SD-STZ and TGR-STZ (table 1).

**Figure 2:**
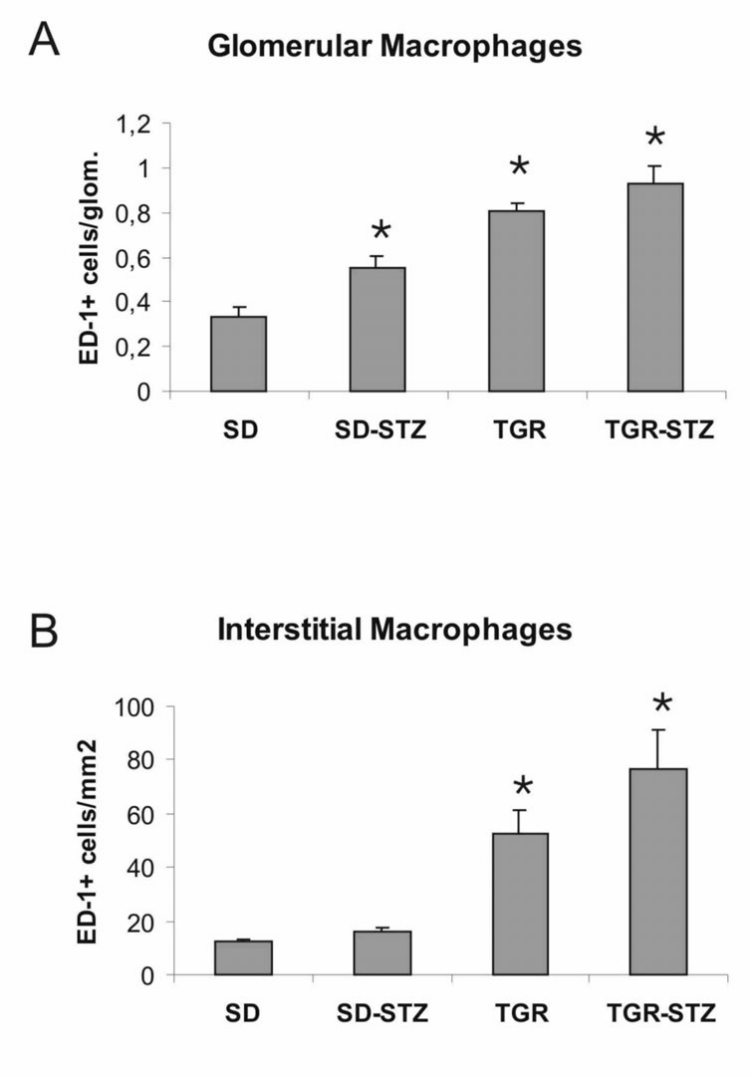
Macrophage infiltration of glomeruli (panel A) and interstitial space (panel B) of the kidney from rats with diabetes mellitus and/or hypertension. Glomerular macrophages are expressed as ED-1-positive cells per glomerular cross-section, interstitial macrophages as ED-1-positive cells per square mm. SD, normotensive normoglycemic control rats; TGR, transgenic hypertensive rats; STZ, streptozotocin treatment. Data are mean ± SEM of n = 5 rats. * indicates p < 0.05 versus normotensive normoglycemic SD control rats.

Both, diabetes and hypertension caused macrophage infiltration of the kidney (figure 2). The effect of hypertension, however, was more pronounced. TGR-STZ animals had the highest levels of macrophages in glomeruli but there was no statistically significant difference compared to TGR (figure 2). Minor macrophage infiltration was observed in the interstitial space of SD-STZ rats with diabetes alone while marked macrophage influx was noted in glomeruli of normoglycemic hypertensive TGR (figure 2). In the combined model of diabetes and hypertension in TGR-STZ, the effect of both diseases on macrophage infiltration of the interstitial space appeared to be additive although the numerical difference between TGR and TGR-STZ failed to reach statistical significance (figure 2). Of note, in TGR-STZ rats, interstitial macrophages were often localized in periglomerular areas (not shown).

**Figure 3:**
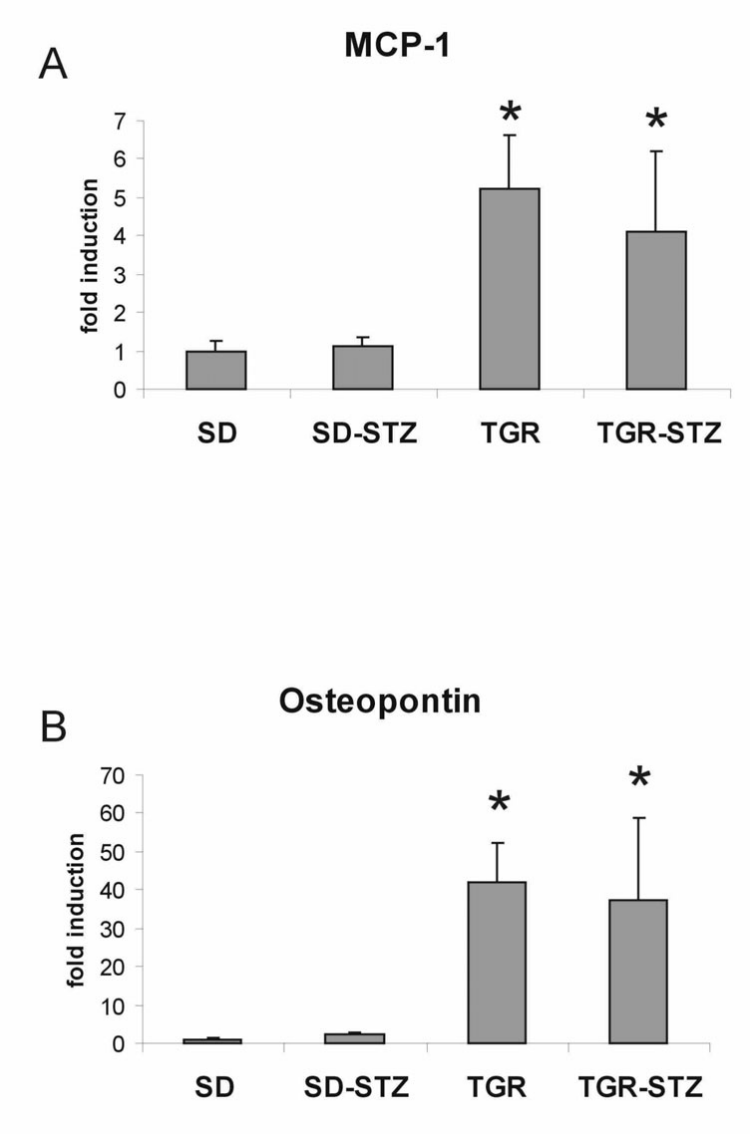
Real-time RT-PCR analysis of MCP-1 (A) and osteopontin (B) mRNA expression in the renal cortex. Data are expressed as fold control compared to SD control rats. Data are mean ± SEM of n = 5 rats. * indicates p < 0.05 versus normotensive normoglycemic SD control rats. SD, normotensive normoglycemic control rats; TGR, transgenic hypertensive rats; STZ, streptozotocin treatment.

Expression of mediators regulating macrophage infiltration (MCP-1, osteopontin, RANTES) and adhesion molecules involved in macrophage infiltration (ICAM-1, VCAM-1) was investigated to further elucidate inflammatory pathways in hypertensive and diabetic renal disease: In TGR rats, renal MCP-1 and osteopontin mRNA steady state levels were increased compared to SD controls, as determined by real-time RT-PCR (figure 3A and 3B). STZ-induced diabetes mellitus of five weeks did not significantly increase MCP-1 and osteopontin mRNA in SD rats and did not augment the upregulation of MCP-1 mRNA in TGR (figure 3). In contrast, the chemokine RANTES or the adhesion molecules ICAM-1 and VCAM-1 were not transcriptionally regulated in the kidney in response to hypertension or diabetes (table 2). The expression level of MCP-1 correlated with macrophage counts in the interstitial space (r^2 ^= 0.47, p = 0.002) but not in glomeruli (r^2 ^= 0.002, p = 0.857).

**Figure 4:**
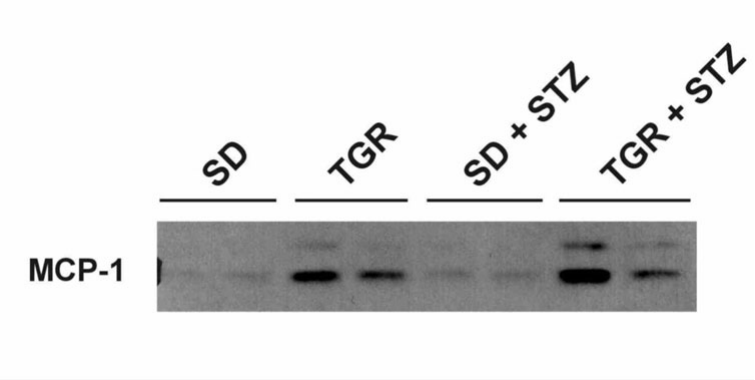
Western blot analysis for MCP-1 protein in cortical protein preparations of two rats of each experimental group, yielding the characteristic MCP-1 double band (14 and 16 kDa). SD, normotensive normoglycemic control rats; TGR, transgenic hypertensive rats; STZ, streptozotocin treatment.

**Figure 5:**
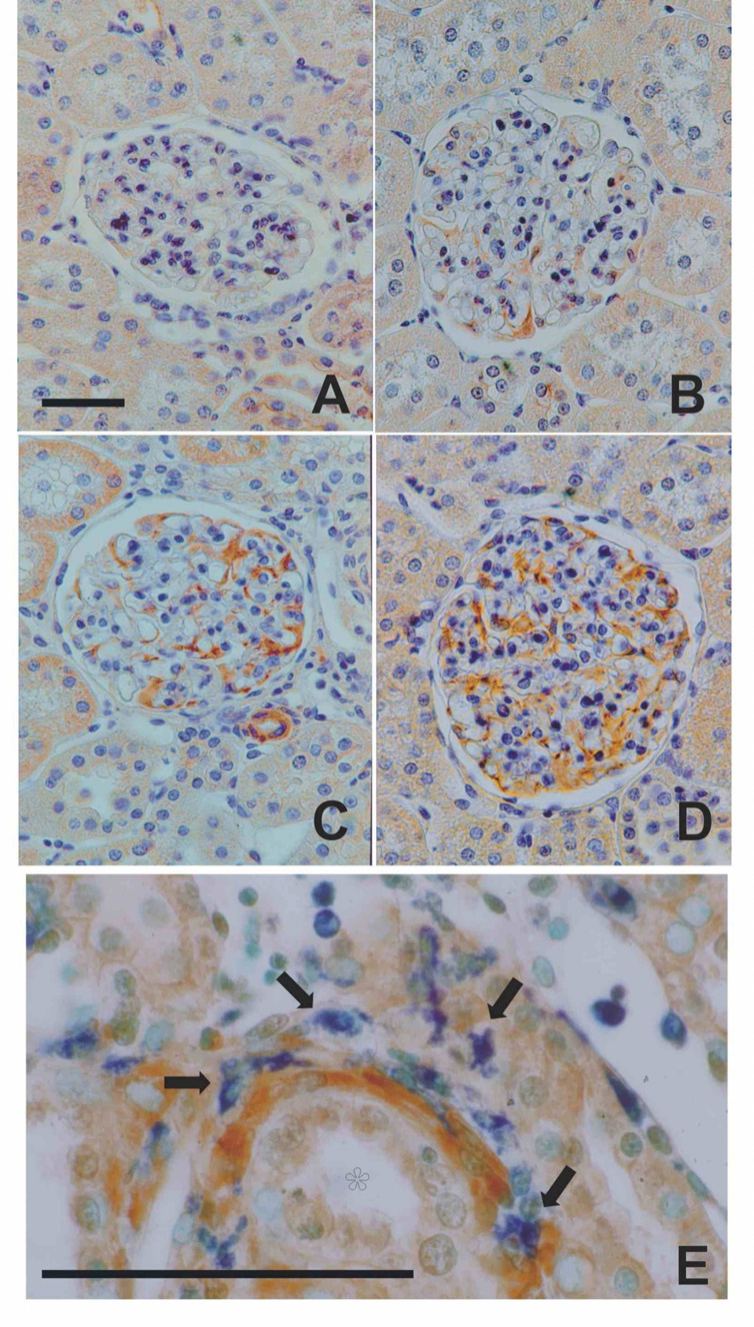
Immunohistochemistry for MCP-1 and the macrophage marker ED-1. Panel A-D: examples of photomicrographs of MCP-1 staining (brown) in glomeruli, hematoxylin counterstain (blue nuclei) Panel A (glomerulus of a normotensive normoglycemic rat) represents score 0, panel B (from a normotensive diabetic rat) score 1, panel C (from a hypertensive normoglycemic rat kidney) score 2, and panel D (from a hypertensive diabetic rat) score 3. Panel E, High power magnification of MCP-1 immunohistochemistry (brown) in a hypertensive diabetic rat. MCP-1 staining localized to spindle-shaped cells, probably fibroblasts, surrounding tubules in a double immunohistochemistry with the macrophage marker ED-1 (blue cytoplasm, arrows), methyl-green counterstain. Macrophages were often localized in close proximity to the MCP-1 positive cells surrounding tubules (asterisk). Scale bars (50 μm) are indicated. Note the identical scale for panels A-D but different scale for panel E.

**Figure 6:**
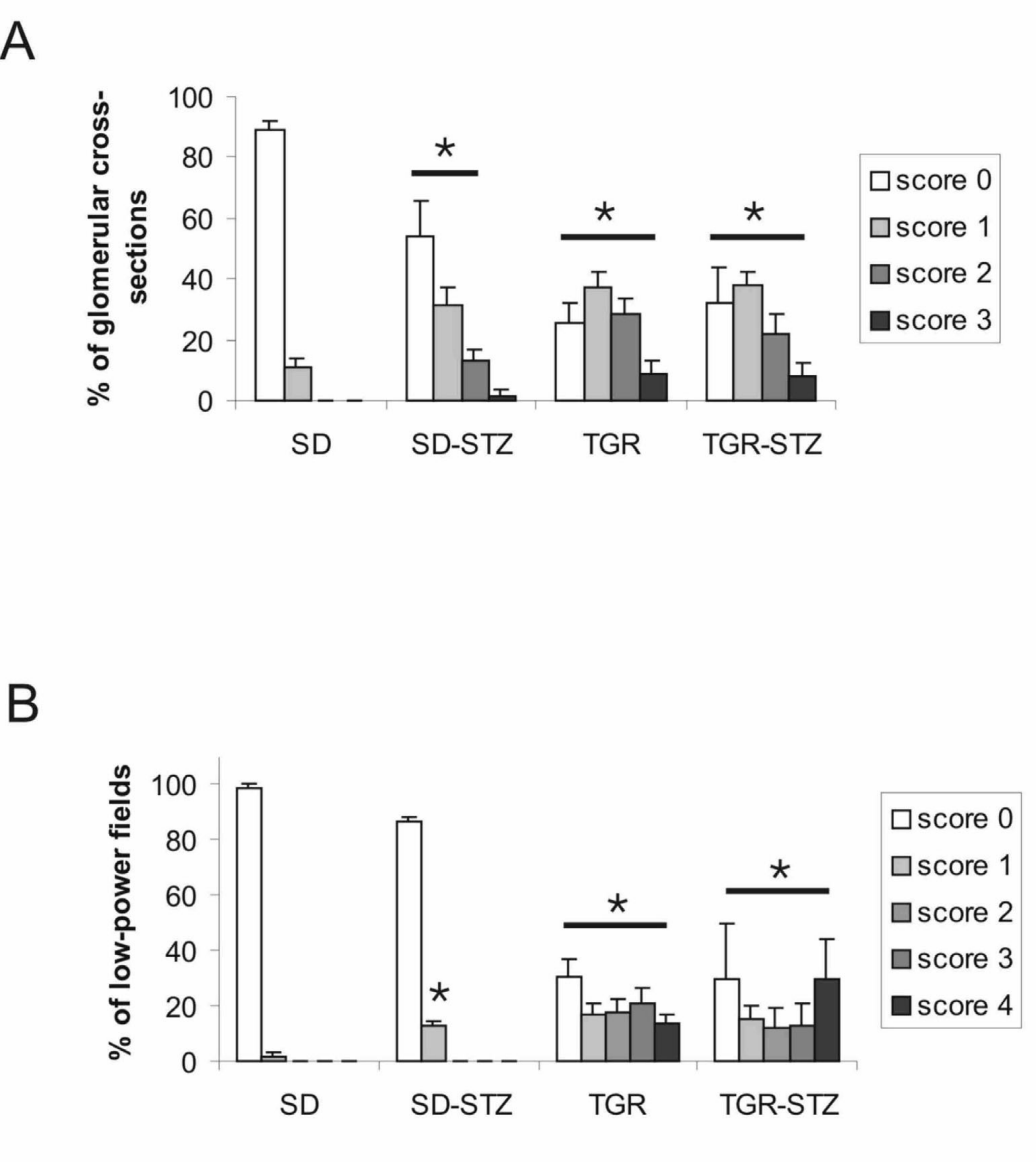
Semiquantitative evaluation of MCP-1 immunohistochemistry. For glomerular staining (A) more than 100 glomeruli per kidney section were classified 0 to 3 (see methods and figure 5 for details). The percentage of glomeruli assigned to a given score value is shown. For interstitial MCP-1 scores (B), 20 low-power cortical fields were classified 0 to 4 (see methods for details). The percentage of interstitial fields assigned to a given score value is shown. SD, normotensive normoglycemic control rats; TGR, transgenic hypertensive rats; STZ, streptozotocin treatment. Data are mean ± SEM of n = 5 rats. * indicates p < 0.05 versus normotensive normoglycemic Sprague-Dawley (SD) control rats.

The specificity of the antibody to MCP-1 was confirmed by Western blot analysis of renal protein (figure 4) yielding a characteristic double band. By immunohistochemistry (figure 5), staining of the smooth muscle layer of small arteries and afferent arterioles was present in controls as well as in diseased animals, albeit more markedly in hypertensive rats (figure 5C). However, almost no glomerular MCP-1 staining was observed in control animals (figure 5A). Focal and segmental positive staining for MCP-1 was detected in glomeruli of normotensive SD-STZ rats with diabetes mellitus (figure 5B). Widespread glomerular staining for MCP-1 (figure 5C and 5D) was seen in TGR and TGR-STZ. By high power light microscopy and double-staining for ED-1, it was noted that intrinsic glomerular cells, rather than infiltrating cells, stained positively for MCP-1. The pattern of immunostaining suggested a predominantly podocyte and occasionally endothelial localization of MCP-1 (figure 5). In the cortical interstitium, MCP-1-staining was localized to peritubular spindle-shaped interstitial cells, possibly fibroblasts, in close proximity to ED-1-positive macrophages (figure 5E). Quantification of glomerular and interstitial MCP-1 staining demonstrated a mild increase in SD-STZ rats and a massive increase in TGR and TGR-STZ (figure 6A). Interstitial staining was little affected by STZ diabetes but markedly induced by TGR hypertension (figure 6B).

**Figure 7:**
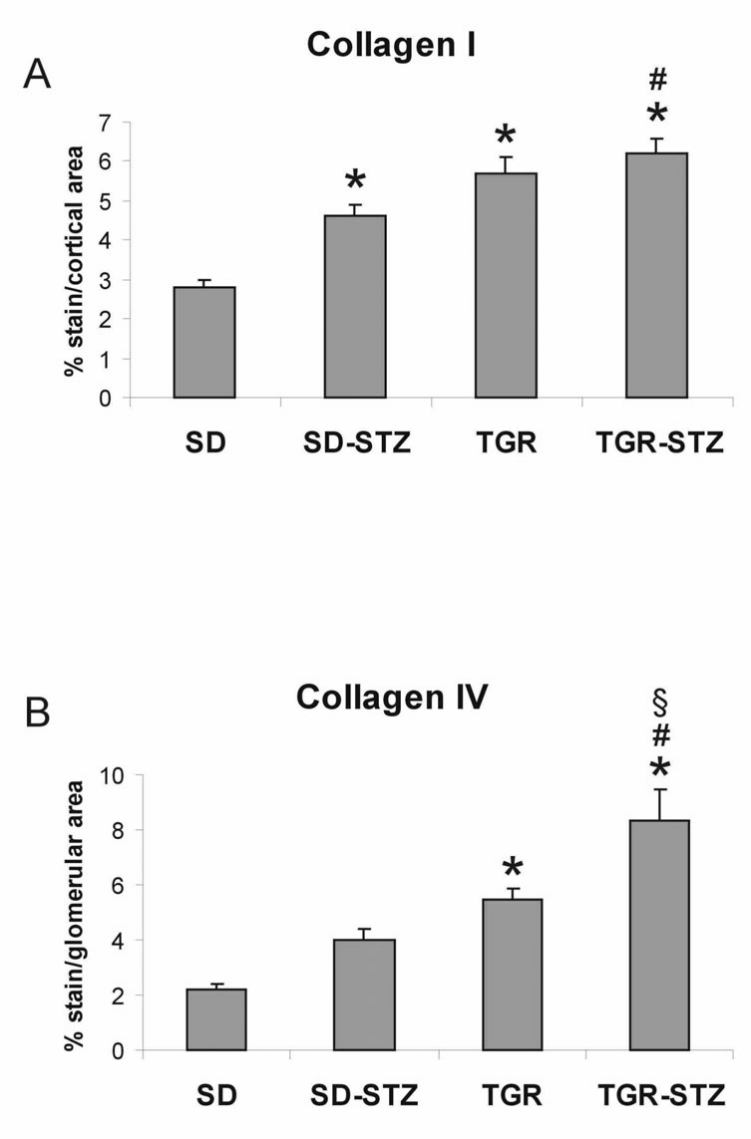
Matrix expansion in the renal cortex and the glomerulus. A: measurement of cortical collagen I staining and B: measurement of glomerular collagen IV staining. Data are mean ± SEM, * indicates p < 0.05 versus normotensive normoglycemic SD controls. # indicates p < 0.05 versus SD-STZ. § indicates p < 0.05 versus normoglycemic TGR.

A moderate matrix expansion was detected in SD-STZ rats in the renal cortex (figure 7A) and in glomeruli (figure 7B) as compared to SD. In TGR, cortical and glomerular matrix expansion was more prominent than in STZ (figure 7A+B), which was further aggravated in TGR-STZ with regard to glomerular matrix (figure 7B) but not to cortical matrix expansion (figure 7A).

## Discussion

The results of the present study confirm that in the rat, both hypertension and diabetes mellitus induce macrophage infiltration in the kidney which may contribute to the development of glomerular and interstitial injury. Moreover, our results confirm and extend previous reports of increased MCP-1 expression in the kidney in diabetes [8-12] and in hypertension [3]. In our study, the effect of hypertension on MCP-1 and osteopontin expression as well as on macrophage infiltration was much more prominent than the effects of diabetes mellitus. Streptozotocin diabetes induced some predominantly glomerular MCP-1 expression and macrophage infiltration whereas hypertensive rats exhibited marked interstitial and glomerular inflammation. This finding cannot be explained by the longer duration of hypertension, compared with diabetes. In preliminary experiments (data not shown), we assured that macrophage infiltration in the kidney is highest from 2 to 6 weeks after STZ injection and decreases thereafter, as described previously by others [7,12]. A different time course of macrophage influx, with a prominent late infiltration, has been found in other rodent models of diabetes, for example in db/db mice with type 2 diabetes [11].

Our data did not reveal evidence for a synergistic effect of TGR hypertension and STZ diabetes on kidney inflammation. Macrophage infiltration tended to slightly higher values in hypertensive, hyperglycemic rats, compared to hypertensive, normoglycemic animals but this trend did not reach statistical significance. These results contrast sharply with those reported by Kelly et al. [13] who described very little inflammation in normoglycemic TGR but a very marked macrophage infiltration in diabetic TGR. We cannot fully explain this discrepancy but several factors may contribute to it. Kelly et al. did not include normotensive controls, neither normoglycemic nor hyperglycemic [13]. Therefore, these authors may have missed the effect of TGR hypertension alone on macrophage infiltration. The shorter duration of diabetes in our study is unlikely to explain the different findings, as discussed above, but the different age and gender of the rats may play a role. We induced STZ diabetes in adult male TGR whereas Kelly et al. employed adolescent (6 week old) female TGR [13]. In other respects, our data confirmed the notion that TGR hypertension aggravates the renal sequelae of STZ diabetes, as described by Kelly et al. [14]. Glomerular sclerosis, as assessed by quantification of collagen IV staining, was significantly higher in hypertensive, diabetic TGR, compared to all other groups.

Real-time RT-PCR was used to screen for large differences of the expression of several proinflammatory molecules, and we focused on the factors which exhibited a clear induction in diabetic, hypertensive TGR. The apparently "negative" results on ICAM-1, VCAM-1 and RANTES should not be misinterpreted to exclude a role of these factors. A more subtle investigation might have shown localized induction of these factors which has in fact been described in hypertensive and/or diabetic kidney disease [4,12,19,20]. The most prominent induction was detected for osteopontin and MCP-1. We focused on MCP-1 because the expression of osteopontin in the diabetic TGR had already been investigated by Kelly et al. [13]. Real-time RT-PCR did not detect an induction of MCP-1 in total cortical RNA of kidney of normotensive, diabetic rats. However, a more subtle investigation by means of immunohistochemistry clearly showed that MCP-1 was increased in glomeruli of normotensive, diabetic rats, in agreement with previous reports [8-12]. MCP-1 was more markedly induced in hypertensive rats but the effects of hypertension and diabetes were not additive. Interestingly, glomerular MCP-1 was localized in podocytes, similar to the localization of osteopontin in glomeruli of diabetic TGR described by Kelly et al. [13]. These findings underscore the potential role of podocytes for glomerular inflammation.

## Conclusion

In our rat model, the effects of hypertension and/or angiotensin II on the expression of the chemokine MCP-1, and on macrophage infiltration, in the kidney were much more pronounced than the effects of diabetes. Furthermore, the effects of both diseases combined on kidney inflammation were not synergistic, and often not even additive. We speculate that the mechanical or hormonal factor – i.e., angiotensin-dependent hypertension – plays a much greater role for the induction of an inflammatory reaction in the kidney than does the metabolic factor, hyperglycemia. In patients with diabetic nephropathy, the decisive contribution of hypertension to renal damage has often been noted [1,21]. Investigators have combined STZ diabetes with genetic models of hypertension, and acceleration of diabetic nephropathy was reported regardless of whether spontaneously hypertensive rats [22], Dahl salt-sensitive rats [23] or mRen2-transgenic hypertensive TGR [14] were used. Our data do not support the notion that inflammation explains this acceleration of diabetic kidney damage because chemokine expression and macrophage infiltration were largely determined by the presence of hypertension, regardless of whether the animals were diabetic or normoglycemic. Other processes, for example accumulation of extracellular matrix, could contribute to the accelerated kidney damage caused by diabetes mellitus in combination with hypertension.

## Competing interests

The author(s) declare that they have no competing interests.

## Authors' contributions

A.H. and K.F.H. drafted this manuscript. K.F.H. and R.V. planned and designed the study. N.C. and M.W. performed and evaluated the animal experiments. A.H., N.C. and M.W. performed and evaluated the histological, molecular biology and immunohistochemical studies. N.C. and M.W. revised the manuscript for intellectual content. All authors have approved the final version of the manuscript.

## Pre-publication history

The pre-publication history for this paper can be accessed here:



## Figures and Tables

**Table 1:** Body weight, organ weights and urine production

**Group**	**SD**	**SD+STZ**	**TGR**	**TGR+STZ**
**N**	5	7	7	6

**Body weight (g)**	429.6 ± 9.0	308.0 ± 9.5 *	388.3 ± 14.6 *	316.3 ± 36.4 * §
**Heart weight (g)**	1.53 ± 0.06	1.24 ± 0.05 *	1.94 ± 0.06 *	1.47 ± 0.18
**Heart weight / body weight ratio(mg/g)**	3.55 ± 0.11	4.03 ± 0.09 *	5.03 ± 0.19 *	4.63 ± 0.16 *
**Kidney weight (g)**	1.30 ± 0.03	1.53 ± 0.02 *	1.47 ± 0.06 *	1.67 ± 0.13 *
**Kidney weight / body weight ratio(mg/g)**	3.04 ± 0.05	4.98 ± 0.15 *	3.82 ± 0.18 *	5.43 ± 0.33 * §
**Urine production (ml/24 h)**	22.0 ± 2.7	95.7 ± 10.1 *	30.0 ± 1.7 *	111.5 ± 13.6 * §

* p < 0.05 versus SD

§p < 0.05 versus TGR

**Table 2:** mRNA expression of the chemokine RANTES and the adhesion molecules ICAM-1 and VCAM-1.

	**SD**	**SD-STZ**	**TGR**	**TGR-STZ**
**RANTES**	1.0 ± 0.26	0.57 ± 0.03	0.47 ± 0.07	0.6 ± 0.12
**VCAM-1**	1.0 ± 0.14	0.98 ± 0.33	1.34 ± 0.51	0.65 ± 0.16
**ICAM-1**	1.0 ± 0.25	1.31 ± 0.49	0.95 ± 0.32	0.68 ± 0.23

Data are fold induction relative to SD as assessed by real-time RT-PCR from renal cortex and are displayed as mean ± standard error of the mean. SD Sprague-Dawley Hannover rats. STZ streptozotocin-treated. TGR Ren-2 transgenic rats. There were no significant differences.
